# Clinical and chest computed tomography features of patients suffering from mild and severe COVID-19 at Fayoum University Hospital in Egypt

**DOI:** 10.1371/journal.pone.0271271

**Published:** 2022-07-08

**Authors:** Ahmed Ismail, Ahmed S. Doghish, Walid F. Elkhatib, Ahmed M. Magdy, Eman E. Mahmoud, Mona I. Ahmed, Mahmoud A. F. Khalil

**Affiliations:** 1 Department of Biochemistry, Faculty of Pharmacy (Boys), Al-Azhar University, Nasr City, Cairo, Egypt; 2 Department of Biochemistry, Faculty of Pharmacy, Badr University in Cairo (BUC), Badr City, Cairo, Egypt; 3 Microbiology and Immunology Department, Faculty of Pharmacy, Ain Shams University, African Union Organization St., Abbassia, Cairo, Egypt; 4 Department of Microbiology and Immunology, Faculty of Pharmacy, Galala University, New Galala City, Suez, Egypt; 5 Department of Radiology, Faculty of Medicine, Fayoum University, Fayoum, Egypt; 6 Department of Clinical and Chemical Pathology, Faculty of Medicine, Fayoum University, Fayoum, Egypt; 7 Department of Chest Diseases, Faculty of Medicine, Fayoum University, Fayoum, Egypt; 8 Department of Microbiology and Immunology, Faculty of Pharmacy, Fayoum University, Fayoum, Egypt; Sam Higginbottom University of Agriculture, Technology and Sciences, INDIA

## Abstract

**Background:**

In pandemic COVID-19 (coronavirus disease 2019), the prognosis of patients has been determined using clinical data and CT (computed tomography) scans, but it is still unclear whether chest CT characteristics are correlated to COVID-19 severity.

**Aim:**

To explore the potential association between clinical data and 25-point CT score and investigate their predictive significance in COVID-19-positive patients at Fayoum University Hospital in Egypt.

**Methods:**

This study was conducted on 252 Egyptian COVID-19 patients at Fayoum University Hospital in Egypt. The patients were classified into two groups: a mild group (174 patients) and a severe group (78 patients). The results of clinical laboratory data, and CT scans of severe and mild patients, were collected, analyzed, and compared.

**Results:**

The severe group show high significance levels of CRP, alanine aminotransferase (ALT), aspartate aminotransferase (AST), creatinine, urea, ferritin, lactate dehydrogenase (LDH), neutrophil percent, and heart rate (HR) than the mild group. Lymphopenia, hypoalbuminemia, hypocalcemia, and decreased oxygen saturation (SpO2) were the most observed abnormalities in severe COVID-19 patients. Lymphopenia, low SpO2 and albumin levels, elevated serum LDH, ferritin, urea, and CRP levels were found to be significantly correlated with severity CT score (*P*<0.0001).

**Conclusion:**

The clinical severity of COVID-19 and the CT score are highly correlated. Our findings indicate that the CT scoring system can help to predict COVID-19 disease outcomes and has a strong correlation with clinical laboratory testing.

## Introduction

Globally, as of 22 April 2022, there have been more than 505,817,953 confirmed cases of COVID-19, including more than 6,213,876 deaths, reported to WHO since the disease’s outbreak in December 2019. Severe COVID-19 signs include inflamed lungs, mucus formation in the airways, and elevated levels of proinflammatory cytokines [1]. The respiratory system is the primary target of the COVID-19 virus due to the high expression of the ACE2 (angiotensin-converting enzyme 2), which mediates the attachment followed by internalization of the virus, however other organ systems such as the heart, liver, central nervous system, kidney, and gastrointestinal tract are also affected [2, 3]. COVID-19 has a wide range of respiratory symptoms, from mild to severe hypoxia in patients with ARDS (acute respiratory distress syndrome). The interval between the commencement of symptoms and ARDS development was as little, implying that respiratory symptoms could develop quickly. Fever, dry cough, and dyspnea are some of the signs of a lower respiratory tract infection [3]. In addition, headaches, dizziness, widespread weakness, vomiting, and diarrhea were also reported [4]. In severe COVID-19, the lung is the most damaged organ, and lung damage is the principal cause of mortality in most patients. Edema, epithelial destruction, and capillaritis/endothelialitis are common early alterations that are commonly accompanied by microthrombosis. Patients with evident respiratory insufficiency develop exudative DAD (diffuse alveolar damage) [5]. The GGO (ground-glass opacities) are the most common radiological manifestation of COVID-19 [6]. Also, during the course of the disease, other indications such as consolidation and a crazy pavement appearance occur [7]. The degree of involvement and affection of the lung was detected by the CT (chest computed tomography), which is the critical diagnostic technique for the diagnosis and severity assessment of COVID-19 because of its great sensitivity in displaying lung disorders [8]. The standard gold test for COVID-19 diagnosis is rRT-PCR from nasal or oropharyngeal swabs. However, in hospitals, CT is commonly used as it can give quick results and has a high diagnostic accuracy [9, 10]. Clinical examination and laboratory investigations, in combination with chest imaging, are widely agreed to be critical for proper patient management [11]. A retrospective study of 252 Egyptian COVID-19 patients at Fayoum University Hospital was conducted. The goal of this study was to investigate the impact of CT severity score alone or in combination with laboratory data in the prognostic assessment of COVID-19.

## Methods

### Patients

This study was conducted on 252 Egyptian COVID-19 patients at Fayoum University Hospital (Fayoum, Egypt) from 1 March 2021 to 25 April 2021.

### Study design

The study started with COVID-19 detection by RT-PCR using nasopharyngeal swabs taken by a health care worker. Then Blood samples were taken from all study patients and used for biochemical and hematological testing. Also, this study involved data collection for clinical and laboratory information for all patients, CT acquisition, and analysis, followed by classification of COVID-19 patients into groups according to the CT severity score. Furthermore, the association between CT severity scores and clinical and laboratory data was also analyzed.

This study was conducted Under the ethical principles of the 1975 Declaration of Helsinki and approved by the Ethics Committee of Fayoum University Hospital. Before any research activity, each patient gave written informed consent to the use of anonymized patient data in scientific research at the time of admission.

### Data collection

Using standardized forms for all patients, clinical and laboratory information including clinical symptoms and vital signs [fever, dry cough, fatigue, dyspnea, myalgia, diarrhea, nausea, headache, vomiting, loss of smell and loss of taste, sore throat, heart rate (HR), body temperature, respiratory rate (RR), and oxygen saturation (SpO2)], routine blood tests [hemoglobin, total leukocyte count (TLC), red blood cell (RBC) count, lymphocyte %, lymphocyte count, neutrophil %, monocyte %, monocyte count, staff cells, and platelet count], blood biochemistry tests including liver and kidney function testes [aspartate aminotransferase (AST), albumin, alanine aminotransferase (ALT), total and direct bilirubin, creatinine, and urea], electrolytes [sodium (Na), and potassium (K)], calcium (Ca), the inflammatory marker C-reactive protein (CRP), thrombotic activity (D-dimer), coagulation profile [prothrombin time (PT), prothrombin concentration (PC), and international normalized ratio (INR)], serum lactate dehydrogenase (LDH), and ferritin were collected from electronic medical records from all patients.

Complete blood count (CBC) was measured using Sysmex Xn 1000 automatic hematology analyzer (Sysmex UK Ltd, UK). The biochemical parameters such as liver, renal function, ferritin, CRP, and LDH were measured using Beckman Au 480 automated clinical chemistry analyzer. (Beckman Coulter, Brea, Calif., USA). Serum PCT was measured using Cobas e 411 (Roche Diagnostics, Mannheim, Germany), and D-dimer and other coagulation tests were performed using Sysmex Cs-1600 (Sysmex UK Ltd, UK). Laboratory tests were performed according to the manufacturer’s instructions. We evaluated our results by daily internal quality control by comparing the measured values with the values obtained from the standard control provided for each medical device, in addition to regular use of UK NEQAS external quality control. Our results are found to be very close to accurate values.

### CT acquisition and analysis

Using a 16-channel CT scanner (Toshiba Medical Systems; Aquilion Lightning), a whole chest CT scan was performed for all patients. Two chest radiologists (with 15 years and 27 years of experience in interpreting chest CT images) independently evaluated all patients, blinded to clinical characteristics and laboratory data. Any disagreement between the two observers was resolved by consensus [12–14]. The presence of parenchymal abnormalities, such as ground-glass opacities (GGO), multifocality, consolidation, distribution (peripheral or diffuse), crazy paving, septal thickening, pleural effusion, pulmonary nodules, and mediastinal lymph nodes, were assessed on CT images of each patient. Additionally, for each lobe, the CT score was assigned as follows: Score 0 for no involvement; 1 for the involvement of less than 5%; 2 for the involvement of 5 to 25%; 3 for the involvement of 25 to 50%; 4 for the involvement of 50 to 75% involvement; and 5 for more than 75% involvement, then multiplied by 5 to get the overall severity score [14]. The total CT severity score, which ranged from 0 to 25, was the sum of each lobe’s points. The cut-off value for severe case identification was 7 [12–14].

### Statistical analysis

The median and interquartile range (IQR) were used to present all the data, and GraphPad Prism (GraphPad Prism 8.0, San Diego, USA) was used to perform all statistical analyses. When more than two groups are being compared, a one-way ANOVA test followed by a Tukey’s multiple comparisons test was used; however, when comparing only two different groups, a Student’s t-test was used. Differences were significant at P-value < 0.05.

## Results

### Patient baseline characteristics

**Fig 1:**
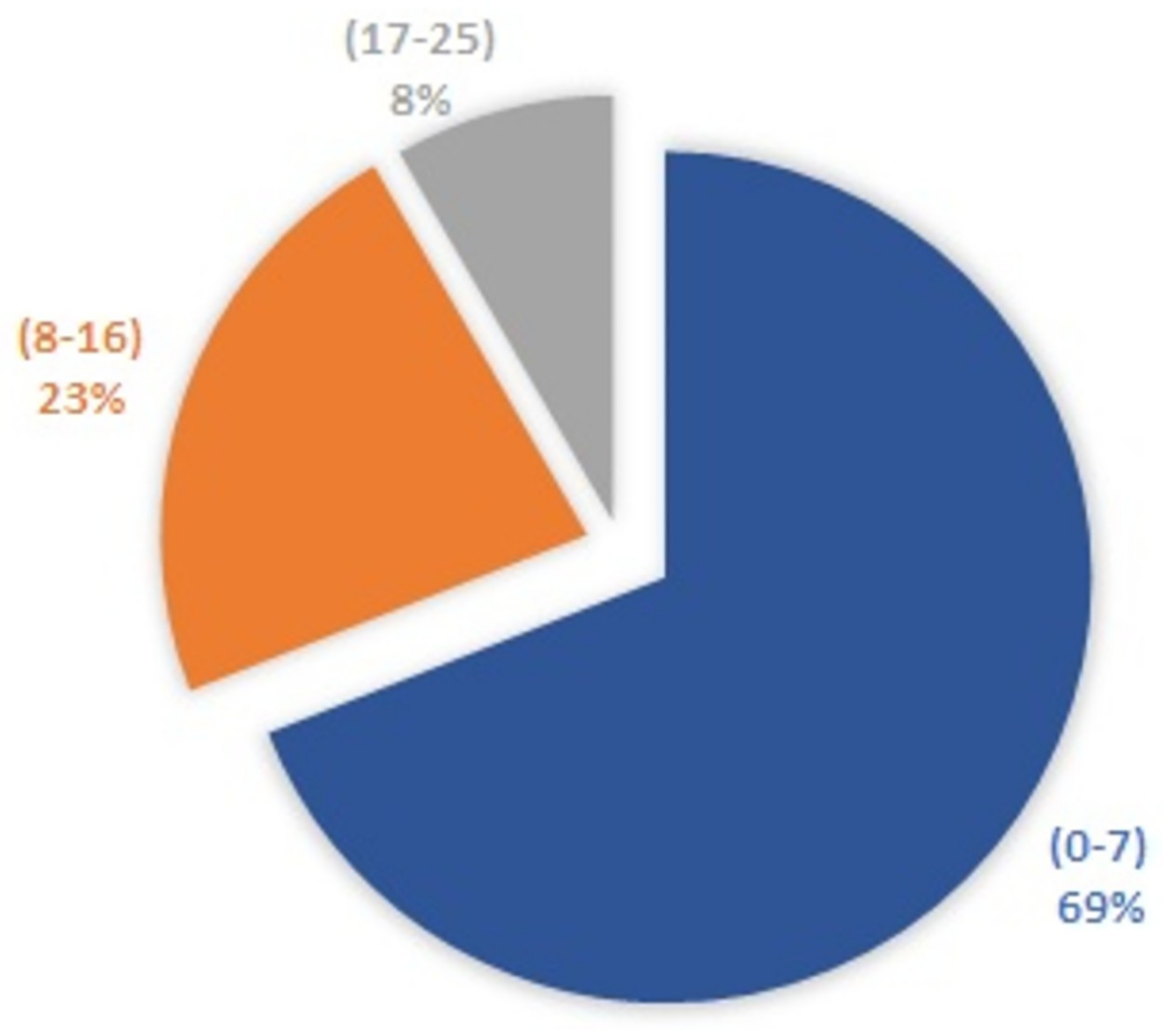
Classification of patients according to 25-point CT severity score. A mild grade is of 0–7 points, a moderate grade is of 8–16 points, and an advanced grade is of 17–25 points. And collectively, as the cut-off value for severe cases identification was 7, the 252 patients in this study were classified into two groups, the mild COVID-19 group (174 patients) with a CT severity score from 0 to 7 points, and the severe COVID-19 group (78 patients) which includes [(moderate grade cases (58 patients) + advanced grade (20 patients)] with a CT severity score of more than 7 points.

In Egypt, at Fayoum University Hospital, 252 patients were diagnosed with COVID-19 and included in this retrospective study. The COVID-19 patients comprising 168 (66.93%) male and 84 (33.47%) female. The median age of the patients was 44.5 years (IQR 35–55.75). Among them, 174 [(69%) (113 male and 61 female)] were diagnosed with mild COVID-19 and CT severity score from 0–7 points and a median of age 44.5 (IQR 34–56) years. In addition, 78[(31%) (55 male and 23 female) including [(58 patients (23%) with moderate severity score (8–16) and 20 patients (8%) with advanced severity score (17–25)] their median age of 44.5 (IQR 35.75–55) years. Due to the cut-off value for severe case identification was 7 [12–14], patients with moderate and advanced CT severity score were enrolled under the severe group [78 (58 moderate + 20 advanced) patients], while the reminder patients with CT severity score 0–7 points were enrolled under the mild group (Fig 1). Thus our study groups are mild (174 patients) and severe (78 patients).

No significant difference (p > 0.05) was observed between the groups regarding the age. In addition, the COVID-19 patients presented with common symptoms of fever (n = 99 [39.29%]), dry cough (n = 73[28.96%]), fatigue (n = 104[41.27%]), dyspnea (n = 63[25%]), myalgia (n = 30[11.9%]), diarrhea (n = 63[25%], nausea (n = 41[16.27%], headache (n = 82[32.54%], vomiting (n = 33[13.1%], loss of smell and loss of taste (n = 51[20.42%], and sore throat (n = 90[35.71%]) were reported.

Fever was found in 39.29% (n = 99) of patients for more than one day. It was significantly higher (*P* < 0.0001) in patients with severe COVID-19 (65.38%, n = 51) than patients with mild COVID-19 (27.59%, n = 48). Among 73 (28.96%) SARS-CoV-2 positive patients. Dry cough was also significantly higher in patients with severe COVID-19 (50%, n = 39) than patients with mild COVID-19 (19.54%, n = 34) at (*P* < 0.0001). The frequency of fatigue, myalgia, dyspnea, diarrhea, nausea, headache, vomiting, loss of smell, loss of taste, and sore throat increased significantly with the severity of the disease (p <0.05). (Table 1).

**Table 1:** Baseline characteristics of patients included in the study.

Item	Total (n = 252)	Mild COVID-19 (n = 174)	Severe-COVID-19 N (n = 78)	*P*-value*
**Age, median (IQR), years**	44.5 (35–55.75)	44.5 (34–56)	44.5 (35.75–55)	0.715
**Sex n (%)**				0.388
Male	168 (66.93)	113 (64.94)	55 (70.51)	
Female	84 (33.47)	61 (35.06)	23 (29.49)	
**Signs and symptoms n (%)**				
Fever	99 (39.29)	48 (27.59)	51 (65.38)	**0.0001**
Dry cough	73 (28.96)	34 (19.54)	39 (50)	**0.0001**
Fatigue	104 (41.27)	60 (34.48)	44 (56.41)	**0.001**
Myalgia	30 (11.9)	8 (4.6)	22 (28.21)	**0.0001**
Dyspnea	63 (25)	24 (13.79)	39 (50)	**0.0001**
Diarrhea	63 (25)	37 (21.26)	26 (33.33)	**0.04**
Nausea	41 (16.27)	11 (6.32)	30 (38.46)	**0.0001**
Headache	82 (32.54)	42 (24.14)	40 (51.28)	**0.0001**
Vomiting	33 (13.1)	9 (5.17)	24 (30.77)	**0.0001**
Loss of Smell	51 (20.24)	26 (14.94)	25 (32.05)	**0.001**
Loss of taste	51 (20.24)	26 (14.94)	25 (32.05)	**0.001**
Sore throat	90 (35.71)	49 (28.16)	41 (52.56)	**0.0002**
D-dimer	127(50.39%)	70 (40.23%)	57(73.07%)	**0.001**

IQR: interquartile range. Data are represented as median (IQR) or n (%).

*All bold values were statistically significant

Thrombotic activity (D-dimer) was found to be significantly higher in severe cases 57(73.07%) than in mild cases (Table 1).

### Computed tomography (CT) findings

**Fig 2:**
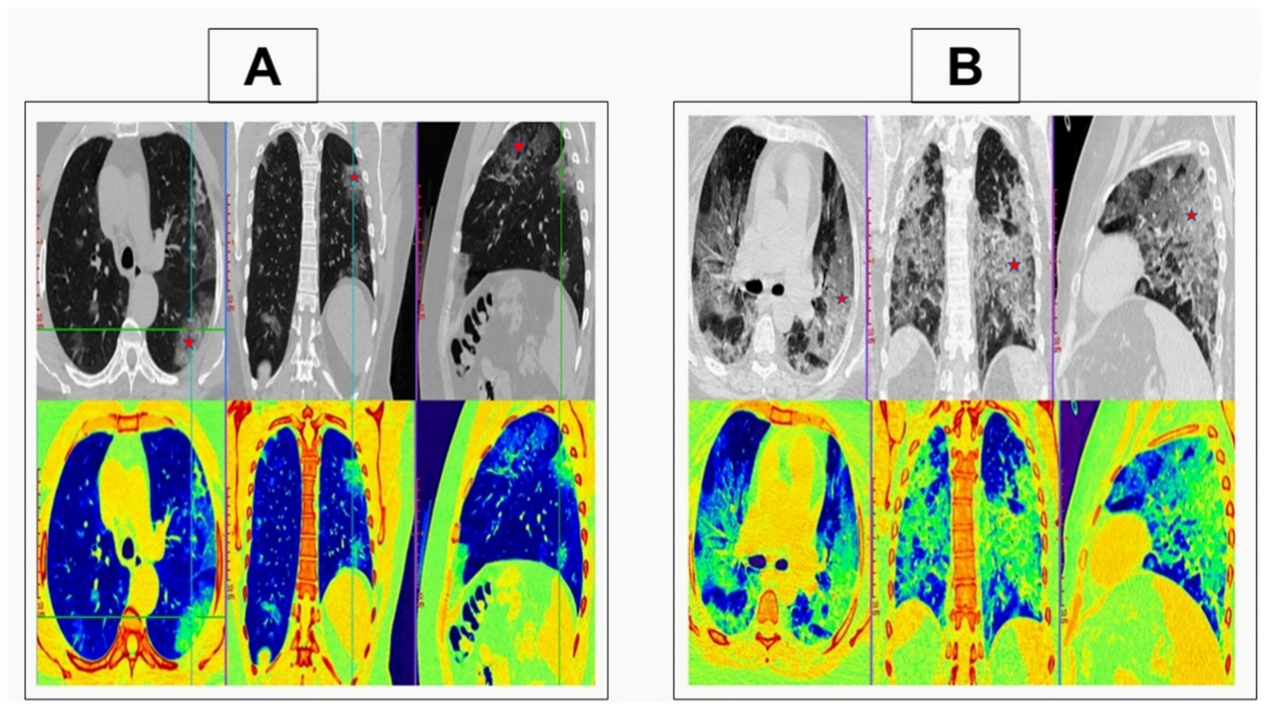
CT scan chest with multi-planar and color-coded images. (A) A 44 years old male patient presented with fever and cough. CT scan chest with multi-planar and color-coded images showed bilateral multi-focal patchy ground-glass opacities with a peripheral subpleural predominance (asterisk). CT severity score was 7. The patient exhibited a mild disease course. (B) A 30 years old male patient presented with fever, dyspnea, and cough. CT scan chest with multi-planar and color-coded images showed widespread bilateral central and peripheral confluent ground-glass opacities (asterisk). CT severity score was 21. The patient exhibited a severe disease course.

Bilateral pulmonary illness was found in 77% of COVID-19 pneumonia cases. The pulmonary illness in 91% of COVID-19 pneumonia cases was predominantly peripheral/subpleural. The most frequently involved lobes are the lower lobes, that are involved in 83% of cases with COVID-19 pneumonia. No significant difference in lung affection between both lungs. GGO is the predominant radiological finding seen in 93% of cases with COVID-19 pneumonia. The consolidation is seen only in 39.5% of cases with COVID-19 pneumonia. Mild pleural thickening was noted in 20% of patients, with mild pleural effusion noted in only 3% of patients. No pulmonary cavitations or significant lymphadenopathy were detected. Fig 2A and 2B show illustrative images of mild and severe COVID-19, respectively.

The patient exhibited a mild disease course were presented with fever and cough. CT scan chest with multi-planar and color-coded images showed bilateral multi-focal patchy ground-glass opacities with a peripheral subpleural predominance (Fig 2A). The patient exhibited a severe disease course were presented with fever, dyspnea, and cough. CT scan chest with multi-planar and color-coded images showed widespread bilateral central and peripheral confluent ground-glass opacities (Fig 2B).

### Association between severity and clinical laboratory data of COVID-19 patients

To explore the potential link between COVID-19 severity and clinical laboratory data the hematological, liver, and kidney function data were collected (Table 2). Our data revealed a more pronounced increase in CRP (48 (mg/dL) [6–104]), ALT (42.6 (U/L) [25.03–81.08]), AST (42 (U/L) [23.5–65.5]), creatinine (1 (mg/dL) [0.7–1.475]), urea (37.5 (mg/dL) [26–59]), ferritin (600 (ng/mL) [250–934]), LDH (357.5 (U/L) [245.5–484.5]), HR (90 (beats/min) [85–100]) and neutrophil % (73[57–85]) in group of patients with severe COVID-19 than patients with mild COVID-19 (p<0.05). On the other hand, there is a significant decrease in albumin (3.5 (g/dL) [2.9–4]), lymphocyte % (18[9.59–30]), and lymphocyte count (1280 (per μL) [695–1797]) in severe group than the mild group. As one of the indicators of severity in COVID-19 patients, hypocalcemia was significantly recorded in severe cases (9.4 (mg/dL) [8.9–10]) than mild cases. Also, no significant differences between mild and severe groups in hemoglobin, monocyte%, monocyte count, RBCs count, TLC, and platelet count. The current results indicate potential association between COVID-19 severity and several clinical laboratory data.

**Table 2:** Clinical laboratory data of patients included in the study.

Item	Total (n = 252)	Mild COVID-19 (n = 174)	Severe-COVID-19 N (n = 78)	*P-* value*
Hemoglobin (g/dL)	13 (12–14)	13 (12–14)	13.20 (12.10–14.05)	0.2270
CRP (mg/L)	8.5 (1.075–57)	6 (0.6–24)	48 (6–104)	**<0.0001**
ALT (U/L)	30.30 (22–53.25)	26.8 (22–43.13)	42.6 (25.03–81.08)	**0.0188**
AST (U/L)	32 (23–49)	30 (21–40)	42 (23.5–65.5)	**0.0004**
Creatinine (mg/dL)	0.9 (0.6–1.2)	0.8 (0.5–1.2)	1 (0.7–1.475)	**0.0289**
Urea (mg/dL)	33.5 (25–50)	30 (23–44.25)	37.5 (26–59)	**0.0080**
Albumin (g/dL)	3.8 (3–4)	3.95 (3–4.1)	3.5 (2.9–4)	**0.0009**
Ferritin (ng/mL)	315 (149.4–600)	232.8 (125–450)	600 (250–934)	**<0.0001**
LDH (U/L)	251 (196–370)	230 (187–319)	357.5 (245.5–484.5)	**<0.0001**
Calcium (mg/dL)	9.8 (9–10.4)	9.9 (9–10.6)	9.4 (8.9–10)	**0.0376**
Lymphocyte %	21.5 (12–35.75)	25 (15–40)	18 (9.95–30)	**0.0022**
Lymphocyte count (per μL)	1378 (900–1949)	1500 (1005–1980)	1280 (695–1797)	**0.0075**
Monocyte%	9 (6.5–12)	10 (8–13)	7 (5.1–12)	0.0634
Monocyte count (per μL)	480 (360.5–661.3)	486 (380–660)	440 (250–676)	0.2042
HR (beats/min)	90 (85–90)	90 (85–90)	90 (85–100)	**0.0103**
RBC Count (Millions/cmm)	4.86 (4.56–5.3)	4.89 (4.56–5.3)	4.81 (4.44–5.215)	0.4443
TLC (thousands/cmm)	6.2 (4.9–8)	6.2 (5–8)	6.2 (4.9–9.5)	0.1209
Neutrophil %	58 (47–76.4)	50 (41.25–62)	73 (57–85)	**0.015**
Platelet count (thousands/cmm)	219 (189–279.8)	218 (189–269)	242 (184.5–324)	0.0569

CRP: C-reactive protein, ALT: alanine aminotransferase, AST: aspartate aminotransferase, LDH: lactate dehydrogenase, HR: heart rate RBC: red blood cell, TLC: leukocyte count, IQR: interquartile range. Data are represented as median (IQR) or n (%).

*All bold values were statistically significant.

### Correlation between CT severity score and clinical laboratory data

**Fig 3:**
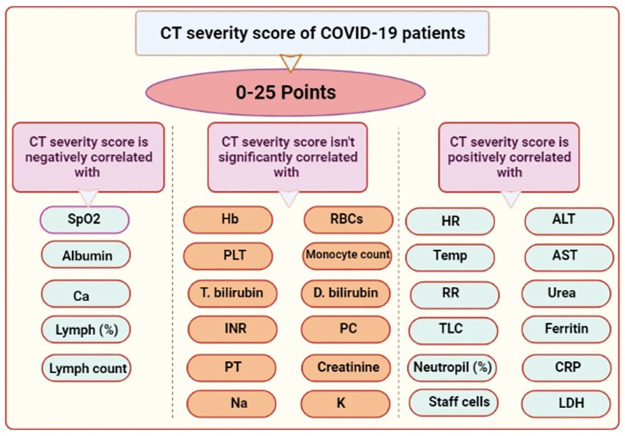
Schematic diagram for the association between different clinical laboratory data and CT severity score in COVID-19 patients.

A correlation analysis was carried out to clarify the potential association between clinical laboratory data and CT severity score in a retrospective study on COVID-19 patients at Fayoum University Hospital in Egypt and their predictive role in disease prognosis. Our data revealed that HR, temperature, RR, TLC, neutrophil (%), staff cells, ALT, AST, urea, ferritin, CRP, and LDH were increased and significantly correlated with CT severity score. Also, SpO2, lymph (%), lymph count, albumin, and calcium were decreased and negatively correlated with CT severity score. On the contrary, Hb, RBCs, PLT, monocytic count, T. bilirubin, D. bilirubin, PT, PC, INR, creatinine, Na, and K were slightly changed but did not significantly correlate with CT severity score (Table 3 and Fig 3). These findings indicate the association between clinical laboratory data and CT severity score in COVID-19 patients at Fayoum University Hospital in Egypt with a good predictive role in prognosis.

**Table 3:** Correlation between clinical laboratory data and CT severity score in COVID-19 patients at Fayoum University Hospital in Egypt.

	CT severity score (points)			Lymph (%)			Ferritin			CRP		
	r	95% confidence interval	*P*-value	r	95% confidence interval	*P*-value	r	95% confidence interval	*P*-value	r	95% confidence interval	*P*-value
**SpO2**	-0.52	-0.6059 to -0.4227	<0.0001	0.19	0.05227 to 0.3293	0.0078	-0.41	-0.5107 to -0.2991	<0.0001	-0.36	-0.4620 to -0.2404	<0.0001
**Hb (gm/dl)**	0.08	-0.06159 to 0.2220	0.2628	0.17	0.03316 to 0.3080	0.0158	0.09	-0.05025 to 0.2321	0.203	0.19	0.04423 to 0.3187	0.0104
**Lymph (%)**	-0.34	-0.4566 to -0.2028	<0.0001	-	-	-	-0.45	-0.5582 to -0.3307	<0.0001	-0.31	-0.4370 to -0.1806	<0.0001
**Urea**	0.16	0.01386 to 0.2939	0.0318	-0.19	-0.3212 to -0.04235	0.0115	0.21	0.07171 to 0.3446	0.0034	0.33	0.1933 to 0.4481	<0.0001
**Creatinine**	0.13	-0.01381 to 0.2677	0.0764	-0.11	-0.2538 to 0.03113	0.1235	0.07	-0.07069 to 0.2126	0.3209	0.21	0.07445 to 0.3456	0.0029
**Ferritin**	0.55	0.4598 to 0.6355	<0.0001	-0.45	-0.5582 to -0.3307	<0.0001	-	-	-	0.65	0.5768 to 0.7209	<0.0001
**CRP**	0.37	0.2566 to 0.4742	<0.0001	-0.31	-0.4370 to -0.1806	<0.0001	0.65	0.5768 to 0.7209	<0.0001	-	-	-
**LDH**	0.34	0.2199 to 0.4447	<0.0001	-0.37	-0.4880 to -0.2419	<0.0001	0.55	0.4499 to 0.6280	<0.0001	0.67	0.5980 to 0.7366	<0.0001
**Calcium**	-0.19	-0.3099 to -0.07036	0.0022	0.09	-0.05412 to 0.2269	0.224	-0.09	-0.2090 to 0.03980	0.1799	-0.16	-0.2841 to -0.04062	0.0096

Notes: Correlation is significant at p<0.05.

## Discussion

COVID-19 was professed a pandemic on 11 March, 2020, by WHO. The clinical spectrum of COVID-19 disease ranges from flu-like symptoms to severe alveolar damage with acute respiratory distress syndrome [15]. To depicts the extent of lung involvement type and degree of parenchymal involvement in COVID-19 patients, a CT chest scan affords information, including GGO and consolidation [16]. However, limited studies have also combined clinical, laboratory, and CT findings. Some of these advocate that the predictive value is improved when adding CT characteristics, while the others show that CT findings had questionable prognostic influence to be used in combination with clinical laboratory data [16–18]. Hence, it could be valuable to understand derangements in COVID-19 by exploring by what means CT severity score associate with clinical laboratory data. Therefore, we aimed to assess the potential connotation flanked by laboratory data and chest severity CT score in COVID-19 patients at Fayoum University Hospital in Egypt.

The results of our study indicated that the COVID-19 patients presented with common clinical symptoms of fever, dry cough, fatigue, dyspnea, myalgia, diarrhea, nausea, headache, vomiting, loss of smell and loss of taste, and sore throat, which were significantly different in mild than severe COVID-19 patients. In the severe group, both the number and degree of symptoms are more obviously remarkable than in the mild group. On the contrary, a previous study indicated that fever, respiratory symptoms, digestive symptoms, and neurological symptoms occurred more frequently in patients with severe COVID-19 pneumonia as compared to that in mild cases [19]. Daozheng Huang et al. examined a large number of COVID-19 patients; they revealed that severe and non-severe patients had distinct differences in clinical symptoms such as diarrhea, vomiting, and dyspnea; however, there were no differences in the symptoms of myalgia, fever, headache, and arthralgia [20]. In addition, the abnormal coagulation marker D-dimer was positive in a large number of severe cases than mild ones, indicating the association between D-dimer and severity in COVID-19 patients. This consequence is described in an earlier study indicating that D-dimer is associated with a severe infection in patients with COVID-19 [21]. Therefore, clinical symptoms of COVID-19 patients had different distribution between severe and mild cases and had a number of implications in predicting and diagnosing severe cases, reducing the mortality, and avoiding COVID-19 more outbreak.

Chest CT scan plays a key role as a diagnostic tool for early detection and management and evaluating the disease severity of COVID-19, especially for false-negative RT-PCR tests [22, 23]. The results of this study indicated that CT features, including bilateral pulmonary affection, predominant peripheral/subpleural distribution of pulmonary affection, GGO, and consolidation were noted in more than 90% of COVID-19 patients, particularly in the severe group including moderate and advanced cases with CT severity score from 8 points to 25 points than the mild group with severity score from 0 points to 7 points.

Moreover, this study indicated the association between CT score and clinical data of COVID-19 patients at Fayoum University Hospital in Egypt, with a good predictive role in prognosis. This proposes that the raise of the vital signs such as HR, temp, RR, and laboratory parameters such as TLC, neutrophil %, staff cells, ALT, AST, urea, ferritin, CRP, and LDH, as well as decreases of SpO2, lymph (%), lymph count, albumin, and calcium, might be helpful in predicting the presence of abnormal CT findings and CT severity. Consequently, the significant association between CT severity score and clinical laboratory data represents the pivotal significance of combining CT and clinical laboratory data, not only in early detection and diagnosis of COVID-19 but also in monitoring the course of disease severity. Collectively, these findings may help in improving health outcomes and achieving less mortality rate. It is noteworthy that the increase in the degree of lung parenchymal involvement and CT severity score is accompanied by decreases in SpO2 due to affected lungs displaying vascular abnormalities, diffuse alveolar injury, and interstitial edema resulting in impaired oxygen exchange [24, 25].

Moreover, there is a potential correlation between CT severity score and inflammatory markers as TLC, neutrophil %, and CRP. These data are in harmony with the previous work performed on the Italian cohort of COVID-19. They observed that the degree of lung parenchymal engrossment was correlated with the raised inflammatory response by increased circulating white blood cells, neutrophil counts, and CRP concentrations [26]. Also, there is a deregulation of the immune response through a massive reduction of lymphocytes which negatively correlates with the disease progression and severity of CT score. Our results are in agreement with previous studies on other populations [26, 27].

Furthermore, the results of laboratory investigations are particularly useful in severe cases to identify damaged organs such as lungs, liver, kidneys, and skeletal muscle. Relevant to previous reports [26, 28], we found that increase in CT severity score was correlated with a significant increase in LDH, ALT, AST, urea levels, and a slight increase in creatinine level. Additionally, a modest decrease in liver synthetic function of albumin with a negative correlation with CT severity score.

Serum ferritin level is generally established to be higher in patients with severe COVID-19 than in those with a mild clinical picture [29–31]; however, the relationship between serum ferritin levels and CT severity score is poorly evaluated. Thus, in our study, we investigated the relationship between serum ferritin levels and CT severity score and the results revealed a significantly positive correlation between serum ferritin level and CT severity score (p < 0.0001). Also, ferritin level correlates significantly with several severity markers other than CT severity score, such as SpO2, lymph %, urea, CRP, and LDH. Accordingly, these data suggest that serum ferritin level is thoroughly related to the COVID-19 patients’ clinical severity as well as radiological severity.

Finally, a previous study indicated that serum calcium is an indicator of COVID-19 and the severe cases had a lower calcium level than mild cases [32]. Nevertheless, and to the best of our knowlege, till now, the mechanism of calcium dysregulation is unclear. However, some researchers believe that the decrease in calcium levels in COVID-19 patients is due to the fact that COVID-19, like many other viruses, has non-structural proteins that can regulate the expression of vasopressin, disrupting the host’s normal ion homeostasis and causing pathological damage such as vomiting, diarrhea, and activation of different inflammatory pathways [33–36]. Others speculated that COVID-19 patients’ low calcium levels were caused by a viral-associated cytokine storm [32]. In addition, calcium was relative to multiorgan injuries, especially in severe cases as calcium plays a pivotal role in physiological processes including deposition of calcium phosphate, neuron electrical signal transmission, platelet function, hormonal regulation, and in several steps of the blood coagulation cascade, subsequently keeping calcium balance was vital to sustaining normal organ functions [32, 37]. Our study found a negative association between blood calcium level and CT severity score, with serum calcium level decreasing as severity CT score increased in COVID-19 patients.

Finally, our study discussed an important topic regarding the association between clinical data and 25 point CT severity score in COVID-19 patients, which might help in diagnosis and management to improve outcomes of COVID-19 patients. But, our study carried out at a single center in Egypt does not represent all Egyptian population, so future studies on a large number on a wide range of the Egyptian population are needed.

## Conclusion

Based on this study, the high level of HR, temp, RR, TLC, neutrophil %, staff cells, ALT, AST, urea, ferritin, CRP, and LDH and low level of SpO2, lymph %, lymph count, albumin, and calcium levels may predict the severity of COVID -19 patients when combined with CT severity score.

## Abbreviations

ACE2: angiotensin-converting enzyme 2
ALT: alanine aminotransferase
ARDS: acute respiratory distress syndrome
AST: aspartate aminotransferase
COVID-19: Coronavirus disease 2019
CRP: C-reactive protein
CT: computed tomography
DAD: (diffuse alveolar damage)
GGO: ground-glass opacities
HR: heart rate
ICU: intensive care unit
INR: international normalized ratio
IQR: interquartile range
LDH: lactate dehydrogenase
PC: prothrombin concentration
PT: prothrombin time
RBC: red blood cell
SpO2: oxygen saturation
TLC: total leukocytes count
